# Identifying Predictors of Early Postoperative Pain and Patient Satisfaction Following Day Surgery: Insights From an Observational Study

**DOI:** 10.7759/cureus.86118

**Published:** 2025-06-16

**Authors:** Salah N EL-Tallawy, Abdullah T Alsubaie, Elsayed A Yousef, Fahriziya T Dahum, Tarek A Abdelzaher, Rania S Ahmed, Vanessa M Alcudia, Walid R Badawy, Sheryll A De Vera, Mohamed S Nagiub, Amal R Aljumah, Essam M Manaa, Wegdan A Ali

**Affiliations:** 1 Anesthesia and Pain Management, King Abdulaziz University Hospital, College of Medicine, King Saud University, Riyadh, SAU; 2 Anesthesia and Pain Management, Faculty of Medicine, National Cancer Institute (NCI) Cairo University, Cairo, EGY; 3 Anesthesia and Pain Management, Faculty of Medicine, Minia University, Minia, EGY; 4 Anesthesia, Anesthesia Department, King Abdulaziz University Hospital, King Saud University Medical City, Riyadh, SAU; 5 Day Surgery Unit, King Khalid University Hospital, King Saud University Medical City, Riyadh, SAU; 6 Anesthesia and Intensive Care, Faculty of Medicine, Minia University Hospital, Minia University, Minia, EGY; 7 Medicine, Alfaisal University College of Medicine, Riyadh, SAU; 8 Anesthesia, King Khalid University Hospital, King Saud University Medical City, Riyadh, SAU; 9 Medicine, College of Medicine, Badr University, Badr City, EGY; 10 Anesthesia, College of Medicine, King Khalid University Hospital, King Saud University, Riyadh, SAU; 11 Anesthesia and Intersive Care, Faculty of Medicine, Minia University Hospital, Minia University, Minia, EGY

**Keywords:** acute postoperative pain, day surgery unit, local anesthetic infiltration, patient’s satisfaction, predictors of postoperative pain, preoperative education, preoperative predictors

## Abstract

Background

Effective management of postoperative pain in the day surgery unit (DSU) is crucial to ensure patient safety and improve satisfaction. The management of postoperative pain in the DSU is challenging due to short hospital stays, variability in the procedures, and physician practices. This study aims to identify factors linked to severe postoperative pain and patient satisfaction levels in the DSU to improve pain management and patient outcomes.

Methodology

This multicenter prospective observational study included 395 patients undergoing different same-day surgical procedures under general anesthesia (GA), regional anesthesia (RA), or combined anesthesia (GA+RA). Data included baseline patient characteristics, anesthetic technique, surgical procedure, interventions like local anesthetic infiltrations, patient education, and pain outcomes. Postoperative pain assessment was done using the Numerical Rating Scale (NRS). Statistical analysis was done to identify the pain outcomes, correlations, and predictors of severe pain and satisfaction. Severe pain is recognized as a pain score (>7/10) by NRS.

Results

The study included 395 patients undergoing different surgical procedures, including Orthopedic (Ortho), obstetrics/gynecology (Ob/Gyn), general surgery (GS), bariatric, urology, and plastic surgeries. Key factors linked to severe postoperative pain included female gender, presence of preoperative pain, and lack of local anesthetic (LA) infiltration. Women experienced more severe pain than men did (55.4% vs. 44.6%). At the same time, men experienced relatively higher satisfaction than women did (6.1 vs. 5.68). LA infiltration reduced worst pain scores (from 6.39 to 5.68) and improved satisfaction (from 5.21 to 6.11). Preoperative education on postoperative pain and pain relief methods reduced worst pain scores (from 6.14 to 5.66) and improved satisfaction (from 5.53 to 6.13). Combined GA+RA provided better pain outcomes than GA or RA alone. Regarding the surgical procedures, Ortho and Ob/Gyn surgeries had the highest incidence of severe pain (40.7% and 40.5%), while urology and plastic surgery patients reported higher satisfaction scores.

Conclusions

Severe postoperative pain in DSU is influenced by gender, preoperative pain, and type of anesthesia. Some interventions, such as LA infiltration, preoperative education, and combined GA+RA, significantly improved pain outcomes and satisfaction. Strategies like procedure-specific postoperative pain management are recommended to enhance pain outcomes and patient satisfaction.

## Introduction

Postoperative pain management is critical to ensuring patient comfort and optimizing early recovery following same-day surgical procedures. It remains a unique challenge in the day surgery unit due to a short timeframe, requiring effective and efficient management of acute postoperative pain, and the expectation of discharge on the same day [1,2]. Inadequate analgesia during the perioperative period is associated with increased morbidity, impaired quality of life, slower recovery, prolonged duration of opioid use, impaired mobilization, delayed discharge, unanticipated hospital admission of day surgery patients, and increased healthcare costs. Research has also shown that inadequate management of acute pain during the perioperative period raises the risk of chronic and persistent pain after surgery [3,4]. Poorly controlled postoperative pain management in the day surgery unit (DSU) is a multifactorial result of inconsistent practices and variability in physician practices, such as poor pain evaluation due to the subjective nature of pain, inadequate patient education, poor patient-provider communication, and inappropriate opioid prescription [3,5,6]. There is a lack of clarity on the division of responsibility regarding the postoperative pain management between nursing staff, anesthesiologists, and surgeons. Nurses are often the first point of contact for assessing and addressing patient pain in the DSU. However, pain management has traditionally been a multidisciplinary effort involving physicians, anesthesiologists, and nursing staff. This division of responsibility can lead to delays in care, inconsistencies in pain management, and patient dissatisfaction [7]. Previous studies indicate that 20%-40% of patients experience moderate to severe pain following day surgery procedures [8]. This high incidence can lead to delayed discharge of the patient, unplanned hospital admissions, and reduced patient satisfaction and quality of life [9,10].

Previous studies have identified several potential predictors of severe postoperative pain, such as demographic factors, psychological factors, clinical and anesthetic factors, and surgical factors [2,8,10]. However, most of these studies have focused on inpatient groups with limited data addressing those predictors in the day surgery units, especially in the Middle East region.

Aim of the study

This study aims to identify predictors of severe postoperative pain and patient satisfaction, such as patient demographics, surgical procedures, anesthesia techniques, and some interventions (e.g., pre-incisional local anesthetic infiltration and preoperative patient education) at the day surgery units of some Middle East hospitals. The findings of this study will be used to identify high-risk patients and allow targeted interventions to reduce the incidence of severe postoperative pain and enhance patient satisfaction at the DSU.

## Materials and methods

Study design

This is a multi-centric prospective observational cohort study. The trial was registered before patient enrollment at clinicaltrials.gov (NCT05624502, registration date: January 1, 2019). The Institutional Review Board (IRB Committee) of the College of Medicine, King Saud University, Riyadh, KSA, has approved this study as a part of the quality improvement program aiming to improve postoperative pain outcomes (Ref. No. 18/0443/IRB). This multi-centric observational study was conducted at the DSUs of three university hospitals (e.g., King Khalid University Hospital (KKUH), King Abdulaziz University Hospital (KAUH), and Minia University Hospital.

Informed written consent was obtained from each participant before enrollment into this study as part of the quality improvement program. Patients then reported their pain scores and side effects related to pain therapy after different surgical procedures. The guideline of Strengthening the Reporting of Observational Studies in Epidemiology (STROBE) was followed in the reporting of the study results [11].

Participants

Patients undergoing same-day surgical procedures under general anesthesia (GA), regional neuraxial anesthesia (RA), or both (GA+RA). The study duration started from January 2020 to May 2024. The study included 395 adults (over 18 years old), male and female patients, with American Society of Anesthesiologists (ASA) physical status I or II, who were undergoing elective same-day surgical procedures. Patients must have an expected minimum stay in the DSU of six up to 12 hours. All patients must be able to provide informed written consent to be included in this quality improvement program. No known allergy or contraindication to the standard pain medications used in the management of postoperative pain was confirmed before inclusion into the study. Exclusion criteria included patients with a history of drug allergy, opioid tolerance, drug abuse, or psychiatric disorders, significant systemic diseases, or diagnosed with cancer were excluded from the study. Additional exclusion criteria related to the study protocol such as patients who refused to participate or were unable to complete the study, those with incomplete documentation, and dropout cases.

Objectives and outcome measures

Primary Objectives

To identify predictors of severe postoperative pain and patient satisfaction with the overall pain management. Severe pain is defined as a pain score >7/10 measured by the Numerical Rating Scale (NRS from 0 to 10) [12]. 

Secondary Objectives

To evaluate the influence of variables such as preoperative predictors (e.g., preoperative pain, LA infiltration, and patient education), type of surgery, and the anesthetic technique on the postoperative pain outcomes (e.g., postoperative pain scores over different time points), worst pain, duration of time on maximum pain and analgesic requirements.

Data collection

Trained nurses from the acute pain services, day surgery unit, or research assistants approached the patients on admission to the day surgery unit. The anesthetic technique, the pain assessment tool, and the methods of postoperative pain relief were explained to all patients before inclusion into the study and during routine immediate preoperative assessments.

Demographic Data

Demographic data include age by year and gender (male or female); surgical procedure (e.g., orthopedic, Ob/Gyn, general surgery, bariatric surgery, urology, and plastic surgery); preoperative patient education; type of anesthesia (e.g., general anesthesia “GA”, regional anesthesia “RA”, or “GA+RA”); pre-incisional local anesthetic (LA) infiltration of the surgical incision site; and intraoperative opioid use (e.g., fentanyl IV dose in mcg).

Pain Outcomes

Pain outcomes include a history of chronic pain during the three months before surgery; immediate postoperative pain score in the Post-Anesthesia Care Unit (PACU); pain on admission to the DSU; and pain scores recorded at 1, 2, 4, 6, and 12 hours postoperatively (or at discharge if the patient was admitted for less than 12 hours). The frequency of severe postoperative pain episodes has been recorded. Additional pain outcomes include worst and least pain scores, the time spent in worst pain, and the patient’s request for more analgesia. Patient satisfaction with the pain management was also completed at the time of discharge from the DSU.

Nursing role in pain management at the DSU

Nurses use a standardized pain management protocol that includes regular pain assessment using tools like the NRS, administration of pre-approved non-opioid medications (e.g., acetaminophen in a dose of 1 g/IV/ 6 hours and NSAIDs, e.g., lornoxicam in a dose of 8 mg/IV/ 8 hours). The administration of controlled medications should be prescribed by physicians. For moderate pain (score 4-6/10 on the NRS), weak opioids such as tramadol (50 mg IV boluses) may be used. For severe pain (score 7-10/10 on the NRS), a low dose of a strong opioid, such as morphine (4 mg IV boluses), may be administered. Nurses assess pain but defer controlled medication decisions and escalations to the physicians, and they maintain continuous communication with physicians if pain control is inadequate.

Sample size

Based on previous studies, a 1-point difference in pain scores on the NRS has been considered clinically significant. Assuming a standard deviation (SD) of 2 points and a power of 80% with a two-sided significance level of 0.05, a sample size of 180-200 patients is enough to provide adequate statistical power and to identify significant predictors for severe postoperative pain. To account for a 10% dropout rate, the target sample size was increased accordingly. Over the 36-month study period (July 2021 to July 2024), a total of 395 patients undergoing same-day surgical procedures under GA, RA, or a combination of both (GA+RA) were enrolled across multiple centers after removal of the excluded and dropout cases. This final sample size exceeds the minimum requirement and ensures adequate statistical power for the planned analyses.

Statistical analysis

The data were analyzed using SPSS Version 21 (IBM Corp., Armonk, NY). Descriptive analyses were conducted to describe demographic characteristics. Categorical variables (e.g., gender, type of anesthesia, and frequency of severe pain) were presented as frequencies and percentages for each category within each procedure group. Continuous variables (e.g., age, preoperative pain score, postoperative pain scores at different time points, and patient satisfaction score) were presented as mean + SD and ranges. Pain scores between groups were compared using an ANOVA test. Correlations between dependent and independent variables are used to explore the relationships between the pain outcome variables (dependent) and the predictor variables (independent). Pearson's correlation coefficient (r) measures the strength and direction of a linear relationship. Spearman's rank correlation coefficient measures the strength and direction of a monotonic relationship (i.e., as one variable increases, the other tends to increase or decrease, not necessarily linearly).

Two regression models were performed: linear regression analysis to identify factors associated with patient satisfaction, including main effects and interaction terms. Logistic regression analysis to identify predictors of severe postoperative pain, including main effects and interaction terms. A *P*-value of <0.05 was considered statistically significant (*P* < 0.05).

## Results

Demographic data

The study included 395 patients who underwent day surgery procedures across six different procedures, e.g., Ortho, Ob/Gyn, GS, Bariatric, Urology, and Plastic surgeries. The baseline characteristics of the study sample are shown in Table 1. The mean age by year is 40.77 ± 11.25, with ages ranging from 22 to 67 years. The data include both male and female patients, with 182 males (46.1%) and 213 females (53.9%). The highest number of participants were orthopedic patients (108, 27.3%), followed by obstetrics and gynecology (Ob/Gyn) patients (79, 20.0%), GS patients (77, 19.5%), bariatric surgery patients (69, 17.5%), and urology patients (32, 8.1%). The lowest number of participants was from plastic surgery (30, 7.6%). Other variables, such as type of anesthesia, preoperative education, and LA infiltration, are shown in Table 1.

**Table 1:** Baseline characteristics of the study groups. ANOVA was used for continuous outcomes (e.g., mean age and intraoperative fentanyl), and chi-square for frequency (e.g., number of patients in each procedure, gender, preop education, type of anesthesia, and LA infiltration). Post hoc tests showed RA vs. GA+RA was significant for severe pain (*P *= 0.02). *P*-value < 0.05 is considered significant (*). ANOVA, analysis of variance; GA, general anesthesia; RA, regional anesthesia; LA infiltration, local anesthetic infiltration

Variables	All procedures	Ortho	Ob/Gyn	GS	Bariatric	Urology	Plastic	Test statistics	*P*-value
Total number	395 (100%)	108 (27.34%)	79 (20.0%)	77 (19.5%)	69 (17.5%)	32 (8.1%)	30 (7.59%)	χ²=5.34	0.0001*
Age (Mean ± SD)	40.77 ± 11.25	41.81 ± 11.05	43.25 ± 12.07	43.64 ± 11.51	35.54 ± 6.65	47.21 ± 11.80	36.91 ± 5.47	*F *= 11.90	0.0432*
Gender (*n*, %)									
Male	182 (46.1%)	61 (56.5%)	0	44 (57.1%)	27 (39.1%)	23 (71.8%)	12 (40%)	*χ*² = 78.3	0.0001*
Female	213 (53.9%)	47 (43.5%)	79 (100%)	33 (42.9%)	42 (60.9%)	9 (28.2%)	18 (60%)		
Preop education (*n*, %)	171 (43.3%)	57 (52.7%)	33 (41.7%)	36 (46.7%)	21 (30.4%)	11 (34.3.8%)	13 (43.3%)	*χ*² =12.1	0.0001*
Anesthesia (*n*, %)									
GA	238 (60.25%)	50 (46.29%)	41 (51.89%)	49 (63.63%)	61 (88,4%)	21 (65.62%)	20 (66.67%)	*χ*² = 45.2	0.0001*
RA	79 (20%)	28 (25.92%)	23 (29.11%)	17 (22.07%)	0	5 (15.62%)	1 (3.33%)		
GA+RA	78 (19.74%)	30 (27.77)	15 (18.98%)	11 (14.28%)	8 (11.6%)	6 (18.75%)	9 (30%)		
LA infiltration (*n*, %)	249 (63.04%)	77 (71.3%)	52 (67.1%)	59 (76.6%)	31 (44.9%)	14 (43.7%)	16 (53.3%)	*χ*² = 24.5	0.002*
Intraop fentanyl (mcg) (Mean ± SD)	158.31 ± 76.25	142.78 ± 79.6	153.73 ± 79.70	158.44 ± 75.82	179 ± 72.5	184.16 ± 85.06	186.36 ± 50.4	*F *= 15.2	0.034*

Pain outcomes and satisfaction based on surgical procedures

Preoperative Pain

**Figure 1:**
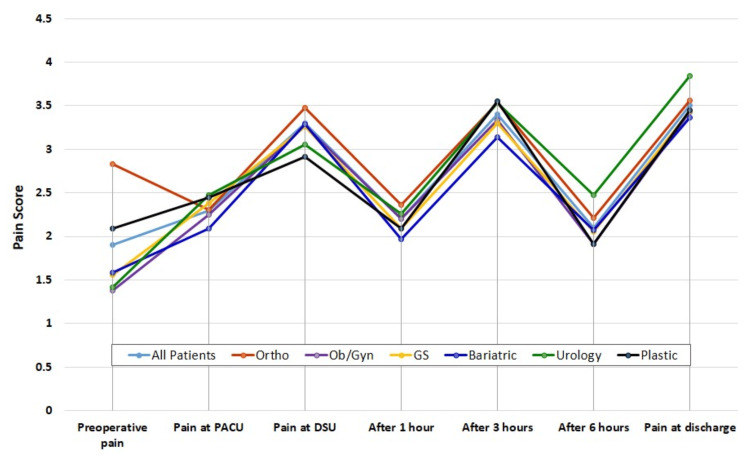
Pain outcomes over time of the study groups. Data are presented as mean pain scores over time. PACU, post-anesthesia care unit; DSU, day surgery unit; GS, general surgery; Ortho, orthopedic; Ob/Gyn, obstetrics/gynecology

The mean of preoperative pain was 1.86, indicating low initial pain levels. The orthopedic surgery patients reported the highest preoperative pain levels (2.83), while Ob/Gyn patients reported the lowest (1.38). Comparison among surgical groups showed highly significant differences (*P* < 0.001) (Table 2; Figure 1).

**Table 2:** Pain outcomes and patient satisfaction of the study groups (Mean ± SD). Time in worst pain was recorded in minutes. ANOVA was used to analyze continuous data (e.g., time in worst pain, postoperative fentanyl, and morphine use), while the chi-square test was used to assess categorical variables such as the incidence of severe pain and the need for additional analgesia. A *P*-value of <0.05 was considered statistically significant (*). PACU, post-anesthesia care unit; DSU, day surgery unit; ANOVA, analysis of variance

Variables	All patients	Ortho	Ob/Gyn	GS	Bariatric	Urology	Plastic	Test statistics	*P*-value
Preoperative pain	1.92 ± 1.62	2.83 ± 1.81	1.38 ± 1.20	1.56 ± 1.53	1.58 ± 1.33	1.42 ± 1.21	2.09 ± 1.64	*F* = 11.90	0.0001*
Pain in PACU	2.29 ± 1.04	2.31 ± 0.98	2.25 ± 1.22	2.38 ± 1.11	2.09 ± 0.95	2.47 ± 0.90	2.45 ± 0.93	*F* = 0.799	0.551
Pain in DSU	3.28 ± 1.33	3.48 ± 1.35	3.27 ± 1.34	3.26 ± 1.43	3.29 ± 1.24	3.05 ± 1.39	2.91 ± 1.45	*F* = 0.723	0.606
After 1 hour	2.15 ± 0.82	2.36 ± 0.83	2.20 ± 0.82	2.09 ± 0.83	1.97 ± 0.80	2.26 ± 0.73	2.09 ± 0.94	*F* = 2.204	0.053
After 3 hours	3.35 ± 1.15	3.53 ± 1.24	3.34 ± 1.05	3.30 ± 1.10	3.14 ± 1.18	3.53 ± 1.47	3.55 ± 0.93	*F* = 1.095	0.363
After 6 hours	2.06 ± 0.78	2.213 ± 0.86	1.91 ± 0.70	2.05 ± 0.79	2.07 ± 0.69	2.47 ± 0.70	1.91 ± 1.04	*F* = 2.431	0.035*
Pain at discharge	3.44 ± 1.21	3.556 ± 1.19	3.43 ± 1.29	3.44 ± 1.22	3.36 ± 1.20	3.84 ± 1.30	3.45 ± 1.04	*F* = 0.583	0.713
Worst pain	5.92 ± 1.53	6.01 ± 1.81	5.95 ± 1.35	5.87 ± 1.41	5.96 ± 1.34	5.47 ± 1.50	6.18 ± 0.87	*F* = 0.495	0.780
Lowest pain	1.77 ± 1.05	1.73 ± 1.02	2.18 ± 1.13	1.74 ± 0.98	1.62 ± 1.4	1.68 ± 0.95	1.45 ± 0.82	F = 2.848	0.015*
Time in worst pain	25.96 ± 12.6	27.59 ± 4.39	25.95 ± 9.17	25.19 ± 14.38	25.58 ± 11.48	24.21 ± 10.17	23.64 ± 9.24	*F* = 0.573	0.072
Severe pain (*n*, %)	146 (36.96%)	44 (40.7%)	32 (40.5%)	23 (29.9%)	25 (36.2%)	10 (31.25%)	11 (36.6%)	*χ*² = 8.3	0.017*
Need analgesia (*n*, %)	164 (41.5%)	42 (38.9%)	34 (43%)	31 (40.3%)	30 (43.5%)	12 (37.5%)	15 (50%)	*χ*² = 9.1	0.021*
Postop tramadol (mg)	21.52 ± 32.01	23.23 ± 34.76	12.03 ± 23.97	25.88 ± 34.26	22.46 ± 32.41	15.79 ± 29.12	25.0 ± 37.08	*F* = 3.87	0.001*
Postop morphine (mg)	2.74 ± 3.32	2.04 ± 3.08	2.35 ± 3.4	2.45 ± 3.34	3.39 ± 3.56	2.74 ± 3.12	2.91 ± 3.51	*F* = 3.87	0.024*
Satisfaction	5.86 ± 2.11	5.67 ± 2.17	5.65 ± 2.11	5.88 ± 2.10	5.72 ± 2.03	6.13 ± 2.63	6.15 ± 1.55	*F* = 0.296	0.915

Pain at PACU

The mean pain score in the post-anesthesia care unit was 2.27, showing a slight increase postoperatively. According to the procedure, the highest pain score was reported for urology (2.47) and plastic surgery (2.45) patients, and the lowest for bariatric surgery patients (2.09). Comparison between the different surgical groups showed no significant differences (*P* = 0.551).

Pain at DSU Admission

The mean pain on admission to DSU was 3.31, suggesting moderate pain levels. Orthopedic patients experienced the highest pain levels upon admission to the DSU (3.48), while plastic surgery patients experienced the lowest (2.91). Comparison among surgical groups showed no significant differences (*P* = 0.606).

Pain at Discharge

The mean pain at discharge was 3.50, indicating moderate pain at discharge. Urology patients had the highest discharge pain scores (3.84), while Bariatric patients had the lowest (3.36). Comparison among surgical groups showed no significant differences (0.713).

Worst Experienced Pain

The mean worst pain was 5.90 for all patients. Plastic surgery patients reported the highest *worst pain scores* (6.18), while urology patients reported the lowest (5.47). Comparison between the study groups showed no significant differences (*P* = 0.780).

Duration of Time in Worst Pain

Orthopedic patients experienced the longest time in maximum pain (27.59 minutes), while plastic surgery patients experienced the shortest time in maximum pain (23.64 minutes). Comparison among surgical groups showed no significant differences (*P* = 0.072).

Incidence of Severe Pain

The highest incidences of severe pain were observed in the Ob/Gyn group (40.5%), while the lowest episodes of severe pain were observed in the GS group (29.9%). Comparison among surgical groups showed significant differences (*P* = 0.017).

Request for More Analgesia

The proportion of patients who needed more analgesia was 40% for all patients, indicating a significant need for additional analgesia. The highest number of patients who requested more analgesia was observed in the Ob/Gyn group (43%), while the lowest number was observed in the urology group (37.5%). Comparison among surgical groups showed significant differences (*P* = 0.021).

Patient Satisfaction

The mean satisfaction score for all patients was 5.73, suggesting moderate satisfaction. Plastic and urology patients reported the highest satisfaction scores (6.16 and 6.13), while Ob/Gyn patients reported the lowest (5.65) (Table 2; Figure 1).

Pain outcomes based on predictors 

Gender

Male patients reported higher satisfaction scores (6.05) compared to female patients (5.55), while the pain scores over time were similar between genders. The incidence of severe pain (34.8% vs. 32.9%) and the need for more analgesia were higher in the female patients compared to males (42.9% vs. 32.9%) (Table 3).

**Table 3:** Pain outcomes and patient satisfaction by some predictors. Time in worst pain was measured in minutes. ANOVA was used to compare pain outcomes between different groups. A *P*-value < 0.05 was considered statistically significant (*). PACU, post-anesthesia care unit; DSU, day surgery unit; ANOVA, analysis of variance; RA, general anesthesia; LA, local anesthetic; GA, general anesthesia

Variable`	All patients (*n*, %)	Pain at PACU (Mean ± SD)	Pain at DSU (Mean ± SD)	Pain at discharge (Mean ± SD)	Worst pain score (Mean ± SD)	Time in worst pain (minutes) (Mean ± SD)	Need more analgesia (*n*, %)	Severe pain (*n*, %)	Satisfaction (Mean ± SD)	Test statistics	*P* – value
Gender											
Male	182 (46.1%)	2.28 ± 0.9	3.35 ± 1.2	3.46 ± 1.1	5.98 ± 1.6	25.38 ± 9.9	65 (32.9%)	65 (44.6%)	6.1 ± 2.0	*F *= 0.01	0.092
Female	213 (53.9%)	2.28 ± 1.1	3.29 ± 1.4	3.48 ± 1.2	5.87 ± 1.4	26.45 ± 14.4	85 (42.9%)	81 (55.4%)	5.65 ± 2.1		
Anesthesia											
GA	238 (60.3%)	2.32 ± 1.1	3.26 ± 1.3	3.49 ± 1.1	5.96 ± 1.4	25.88 ± 12.1	94 (41.2%)	88 (60.2%)	5.71 ± 2.0	*F *= 6.2	0.045*
RA	79 (20%)	2.32 ± 1.0	3.56 ± 1.4	3.55 ± 1.4	5.94 ± 1.9	28.51 ± 15.7	32 (50.8%)	31 (21.2%)	5.66 ± 2.10		
GA+RA	78 (19.7%)	2.06 ± 0.9	3.25 ± 1.2	3.33 ± 1.1	5.91 ± 1.7	23.89 ± 9.0	24 (31.2%)	27 (18.5%)	6.08 ± 1.92		
LA infiltration	249 (63%)	3.26 ± 1.3	3.26 ± 1.3	3.43 ± 1.2	5.68 ± 1.4	25.45 ± 11.9	71 (30.7%)	69 (47.2%)	6.11 ± 2.1	*F *= 5.8	0.016*
No LA infiltration	146 (367%)	3.42 ± 1.3	3.42 ± 1.3	3.55 ± 1.1	6.39 ± 1.4	27.15 ± 13.5	79 (57.7%)	77 (52.8%)	5.21 ± 2.0		
Preop education	171 (43.2%)	2.44 ± 1.1	3.25 ± 1.3	3.40 ± 1.2	5.66 ± 1.4	26.01 ± 12.4	30 (20.1)	37 (24.8%)	6.13 ± 2.1	*F *= 9.1	0.003*
No preop education	224 (56.7%)	2.18 ± 1.3	3.42 ± 1.3	3.58 ± 1.2	6.14 ± 1.5	26.19 ± 11.9	119 (54.6%)	87 (39.9%)	5.53 ± 2.0		

Type of Anesthesia

Patients who received combined GA+RA reported the lowest pain scores, shortest time in worst pain (23.50 minutes), lower incidence of severe pain, less need for more analgesia, and highest satisfaction scores (6.08). Patients who received RA alone experienced the longest time in worst pain (28.51 minutes) and the most requests for additional analgesia. Patients who received GA alone reported the highest incidence of severe pain.

LA Infiltration

Patients who received LA infiltration reported lower worst pain scores compared to those who did not (5.68 vs. 6.39). Patients with LA infiltration also reported significantly higher satisfaction scores (6.11 vs. 5.21). Duration of worst pain was shorter for patients who received LA infiltration (25.45 minutes vs. 27.15 minutes). The other pain scores were lower in patients who received LA infiltration.

Preoperative Education

Patients who received preoperative education reported lower worst pain scores (5.66 vs. 6.14). Patients with preoperative education reported significantly higher satisfaction scores (6.13 vs. 5.53). Pain at PACU was slightly higher for patients who received preoperative education (2.44 vs. 2.18) (Table 3).

Perioperative analgesia

Intraoperative Analgesia

Intraoperative analgesia was administered to all patients. The commonly used analgesics were paracetamol 1 gm IV, followed by the nonsteroidal anti-inflammatory drug (NSAID) lornoxicam 8-16 mg IV as a single dose. Intraoperative fentanyl IV (mean ± SD). Showed wide variations between the patients according to the surgical procedure, with the highest fentanyl consumption reported in plastic (186.36 ± 50.45) and urology patients (184.16 ± 85.06 mcg). The lowest consumption was reported in orthopedic patients (142.78 ± 79.59) (Table 1).

Postoperative Analgesia

Regarding postoperative pain management, all patients received simple analgesics for postoperative analgesia (e.g., paracetamol 1 g IV/6 hours followed by the NSAID lornoxicam 8 mg IV/8 hours). Some patients required additional analgesia due to moderate to severe pain in the form of tramadol 25-100 mg IV (21.06 ± 32.13) for moderate pain and/or morphine in a dose of 2-12 mg IV boluses (2.55 ± 3.32) for severe pain (Table 2).

Correlation analysis 

Preoperative Pain

**Figure 2:**
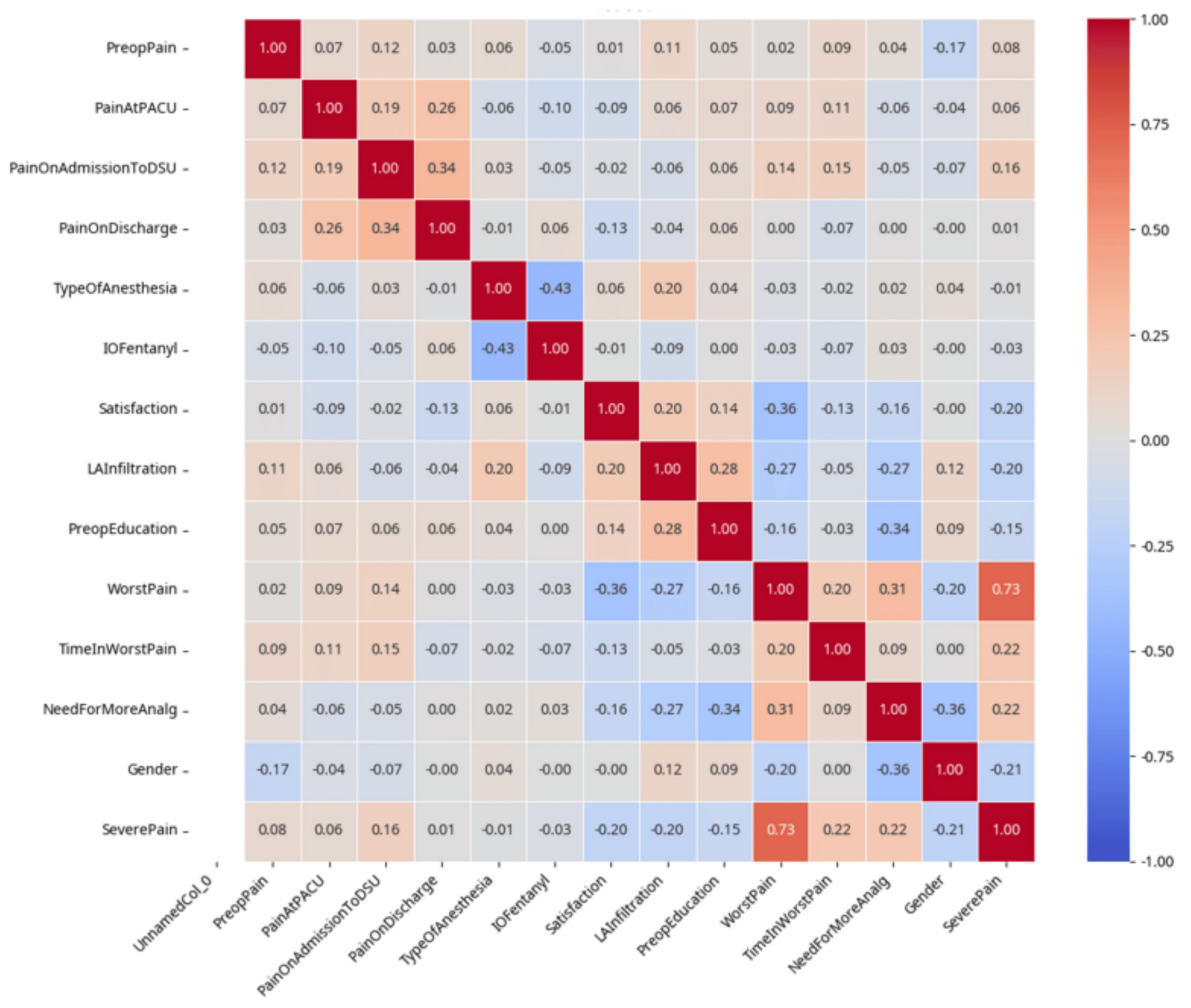
Correlation heatmap results showing linear relationships among various variables. The values range from -1 (perfect negative correlation) to +1 (perfect positive correlation), with 0 indicating no linear correlation. Red indicates a positive correlation, while blue represents a negative correlation. The intensity and shade of the color reflect the strength of the correlation. PACU, post-anesthesia care unit; DSU, day surgery unit

**Figure 3:**
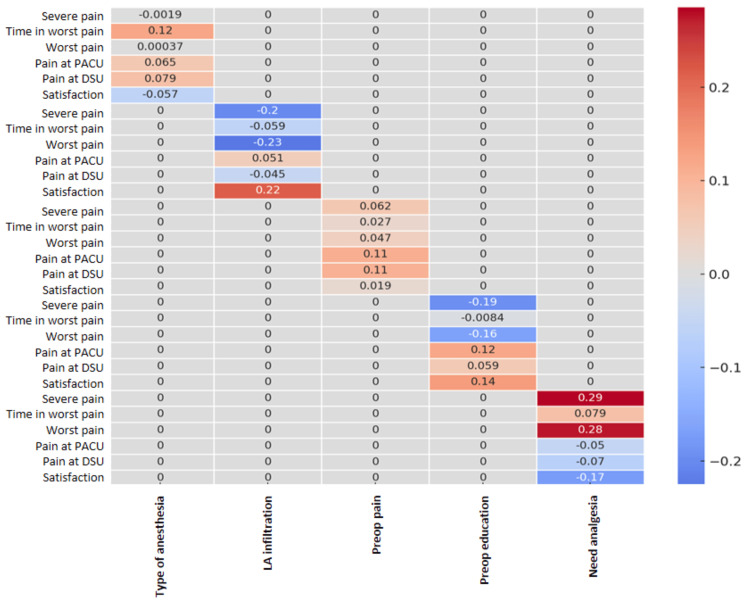
Analysis of correlation heatmap results for selected predictors and study variables. The values range from -1 (perfect negative correlation) to +1 (perfect positive correlation), with 0 indicating no linear correlation. Red indicates a positive correlation, while blue indicates a negative correlation. The intensity and shade of the colors reflect the strength of the correlation. PACU, post-anesthesia care unit; DSU, day surgery unit

Showed weak positive correlations with pain at PACU (*r* = 0.111) and pain at DSU (*r* = 0.107), indicating that patients with higher preoperative pain tend to experience slightly higher pain levels in the immediate postoperative period (Figures 2, 3).

LA Infiltration

LA infiltration showed moderate negative correlations with pain measures, specifically with *Severe pain* (*r* = -0.20) and *Worst pain* (*r* = -0.23). LA infiltration was significantly associated with lower need for additional analgesia (*P *< 0.01) and lower incidence of severe pain (*P *< 0.002).

Preoperative Education

Additionally, preoperative education showed a strong negative correlation with pain measures, specifically with *Severe pain* (*r* = -0.19) and *Worst pain* (*r* = -0.16).

Intraoperative Fentanyl

Showed weak negative correlation with pain at PACU (*r* = -0.100). This suggests that higher doses of intraoperative fentanyl may be associated with slightly lower pain scores in the PACU.

Worst Pain Scores and Frequency of Severe Pain

They showed a strong positive linear correlation (*r* = 0.73). This suggests a meaningful relationship between these two factors. Patients who experienced severe pain had significantly longer duration of worst pain compared to those who did not (*P *< 0.001).

Need for More Analgesia

It has the strongest positive correlations with pain outcome measures, specifically with *Severe pain* (*r* = 0.29) and *Worst pain* (*r* = 0.28). Patients who need more analgesia reported significantly higher worst pain scores.

Pain at Discharge and Satisfaction

The correlation between *pain at discharge* and *satisfaction* is (*r* = -0.13), indicating a weak negative relationship. This weak negative correlation suggests that higher pain levels at discharge do not strongly predict lower satisfaction scores.

Patient's Satisfaction

The satisfaction is positively correlated with *LA infiltration* (*r* = 0.22) and *preoperative education* (*r* = 0.14), but negatively with the *need for more analgesia* (*r* = -0.17). Patient satisfaction was negatively correlated with worst pain scores (*r* = -0.37), indicating that higher pain levels were associated with lower satisfaction (*P *< 0.001) (Figures 2, 3).

Regression analysis 

Linear Regression for Predictors of Satisfaction

The linear regression model for satisfaction showed an *R*^2^ value of 0.243. This result indicates that 24.3% of the variance in satisfaction scores could be explained by these predictors. Significant positive associations were found with LA infiltration and male gender, while higher pain intensity and longer duration of worst pain were negatively associated with satisfaction (Table 4).

**Table 4:** Linear regression for predictors of satisfaction. *P*-value < 0.05 is considered significant. *R*^2 ^= 0.243. Interpretations: 24.3% of variation in satisfaction scores explained by predictors.

Predictor	Coefficient	*P*-value	Significance	Association
Gender (M)	0.542	0.0139	Significant	Positive (males reported higher satisfaction)
LA infiltration (Yes)	0.728	0.0015	Significant	Positive (LA infiltration increased satisfaction)
Worst pain	-0.348	0.0001	Significant	Negative (higher pain scores decreased satisfaction)
Time in the worst pain	-0.027	0.0005	Significant	Negative (longer duration decreased satisfaction)

Logistic Regression Results for Predictors of Severe Pain

The receiver operating characteristic (ROC) curve was used to evaluate the diagnostic performance of the logistic regression model in distinguishing between patients who did and did not experience severe postoperative pain. The logistic regression model for severe pain showed a pseudo *R*^2^ value of 0.596, indicating a strong fit to the data. The model correctly classified severe pain in a significant proportion of cases, with an area under the ROC curve of 0.946, indicating excellent discriminative ability. Predictors of severe pain are significantly associated with worst pain (strong positive association) and time in worst pain (positive association) (Table 5).

**Table 5:** Logistic regression for predictors of severe pain. *P*-value < 0.05 is considered significant. Pseudo *R*^2 ^=0.596. Area under the receiver operating characteristic (ROC) curve: 0.946. Interpretations: Strong model fit with excellent classification accuracy

Predictor	Odds ratio	*P*-value	Significance	Association
Gender (M)	0.67	0.3462	Not significant	Decreased odds of severe pain
LA infiltration (Yes)	0.91	0.8139	Not significant	Decreased odds of severe pain
Worst pain	8.42	0.0001	Significant	Increased odds of severe pain
Time in worst pain	1.03	0.0428	Significant	Increased odds of severe pain

Predictors of severe postoperative pain and satisfaction

Severe Postoperative Pain

Female gender (odds ratio (OR) = 1.87), higher preoperative pain (OR = 1.23) per unit, and lack of LA infiltration (OR = 0.53) are significantly associated with higher rates of severe postoperative pain.

Duration of Time in Worst Pain

A longer duration of worst pain is associated with female gender and higher preoperative pain scores. While LA infiltration is negatively correlated with the time in severe pain.

Satisfaction Score

The satisfaction score is negatively correlated with pain intensity, time spent in worst pain, and orthopedic procedures, while LA infiltration was associated with a higher satisfaction score.

Gender

Worst pain, time in worst pain, and severe pain are higher in females. While the satisfaction score is higher in males.

Preoperative Education

Preoperative education could improve patient outcomes and reduce postoperative analgesia requirements.

Type of Anesthesia

The combination of GA+RA shows the strongest negative correlation between worst pain and satisfaction, followed by RA and GA, suggesting that this combination might lead to lower satisfaction if pain is not well managed.

## Discussion

The management of acute postoperative pain in the DSU remains a critical challenge, considering the implications of the rapid recovery and early discharge from the DSU.

This prospective multi-centric observational study provides a comprehensive assessment of the management of acute postoperative pain, predictors of severe postoperative pain, and satisfaction among patients undergoing same-day surgical procedures. Our findings reinforce the complexity and the challenges of managing early postoperative pain in the DSU.

Pain outcomes in the current study showed wide variations across different surgical procedures and the anesthetic technique. Pain scores generally decreased over time from PACU to discharge, with some variations based on procedure type. Orthopedic patients showed the highest preoperative and early postoperative pain. While urology patients reported higher pain at discharge, at the same time, they reported a relatively higher satisfaction score. According to these outcomes, the study highlights the importance of procedure-specific pain management strategies to target different predictors and causes of postoperative pain based on surgical procedure type. Additionally, there is a significant fluctuation of the pain levels, with notable peaks at specific stages, such as pain levels increasing again at discharge, indicating potential areas for improved pain management, such as the use of multimodal analgesia and the procedure-specific postoperative pain [8]. This approach could reduce the incidence of postoperative pain, improve patient satisfaction, and enhance the quality of life.

Despite the great advances in patient care and management during the perioperative setting, acute postoperative pain remains a major problem due to suboptimal management and presents a significant challenge, especially in the day surgery unit. This is mainly attributed to the short duration of stay and early discharge with limited follow-up of the patient [8,13]. 

Our observations showed that approximately 30%-40% of the patients experienced moderate to severe pain during the early postoperative hours, depending on the type of surgery and the anesthetic approach. These results align with previous studies that reported similar outcomes of unrelieved pain during the first postoperative day [14,15].

Predictors of severe postoperative pain and satisfaction

Our study identified several predictors of severe postoperative pain, including female gender, higher preoperative pain, and lack of local anesthetic infiltration, while also highlighting the benefits of combined GA+RA anesthesia and preoperative education. The current study shows a wide variation in the severity of postoperative pain, with Ob/Gyn and orthopedic procedures associated with the highest rates of severe postoperative pain. This is in agreement with those who observed that orthopedic and Ob/Gyn procedures are among the most painful procedures [16]. This variation in the pain intensity is attributed to many factors, including the extent of tissue trauma, nerve injury, and type of surgery [17-19]. Age had no significant correlation with pain outcomes because all patients admitted to the DSU are healthy with no moderate or severe comorbidities.

Findings of this study identified several key factors influencing the incidence of severe postoperative pain and patient satisfaction. Results revealed that female gender, high preoperative pain, and type of procedure are associated with higher rates of severe pain and reduced patient satisfaction. This aligns with similar studies that found women and preexisting pain are associated with higher postoperative pain [20,21]. Preexisting pain may lead to upregulate of central sensitization and predispose to exaggerated pain experience after surgery [8,21]. 

Pain outcomes did not differ significantly between genders; however, female patients experienced a higher incidence of severe pain, a longer duration of worst pain, and a lower satisfaction score. Male patients reported higher satisfaction scores than female patients. This highlights the potential need for sex-specific pain management, especially in the DSU, where there is not enough time for patients to follow up. These findings align with another review article that analyzed gender differences in postoperative pain and yielded inconsistent findings, with a greater number of women seeming to be at higher risk of developing severe postoperative pain [22]. Similarly, other studies reported that female gender and preoperative pain as consistent predictors of severe postoperative pain. Women are 1.5-2 times more likely to experience higher pain intensity postoperatively, likely due to biological and psychosocial factors, such as differences in pain perception and hormonal influences [23,24]. Preoperative pain is an important factor that significantly predicts postoperative pain, supporting our observation of its weak but positive correlation with early postoperative pain scores [25]. Contrary to these findings, postoperative pain outcomes could not be explained and may be attributed to other factors such as variability in surgical type and analgesic protocols [26].

Role of the interventions

Interventions such as LA infiltration, preoperative education, and type of anesthesia have been confirmed as significant predictors for severe postoperative pain and satisfaction. Both LA infiltration and preoperative education were associated with improved outcomes, suggesting these should be standard practice where possible.

LA Infiltration

It is one of the important findings revealed that some interventions, like LA infiltration before surgical incision, could significantly reduce pain intensity and improve satisfaction. This effect was further confirmed by the negative correlation with both worst pain scores and the incidence of severe pain, in addition to its positive correlation with satisfaction. These findings align with recent findings that reported the effectiveness of LA infiltration as a part of multimodal analgesia in reducing pain intensity following various surgical procedures [27,28]. Routine use of LA infiltration, particularly in orthopedic and gynecologic surgeries, is beneficial for improving postoperative pain outcomes.

In contrast, other studies failed to demonstrate significant effects of LA infiltration, especially in longer and extensive surgeries [29]. In our study, most of the same-day procedures were done within a two-hour duration. This may explain the greater benefit of the LA infiltration observed. 

Preoperative Education

The preoperative education is associated with improved pain outcomes and higher satisfaction scores. Our study revealed that patients who received preoperative education had lower early pain scores and improved satisfaction. This aligns with previous studies that demonstrated the beneficial effects of preoperative education on reducing anxiety, postoperative pain intensity, while improving satisfaction. Standardized preoperative education protocols for all patients are essential [8,30,31].

Anesthetic Technique

The findings of this study showed that the combined use of GA+RA was associated with lower pain scores, lower incidence of severe pain, shorter duration of worst pain, and higher satisfaction score. This aligns with Paladini et al. [2,19,32], who demonstrated that GA+RA reduced pain scores and opioid consumption. A similar study also found that combined GA+RA is associated with improved anxiety, pain outcomes, and reduced opioid consumption after surgery [33,34,35].

However, some studies suggest that RA alone may outperform GA in specific surgeries. It demonstrates superior pain management, improved recovery, and reduced opioid consumption due to more effective nerve blockade [34,36,37]. While Niyonkuru et al. [38] found no significant advantage of GA+RA over GA alone in minor procedures. Our data showed that RA alone was associated with prolonged duration of worst pain. This may be attributed to the titration to the lowest effective dose of LA and no catheter technique, so the block wears off early. This is mainly based on the trend of early ambulation and rapid discharge from the DSU.

Limitations, strengths, and future directions

Limitations

The current study is subject to the limitations, including confounding factors such as variations in surgical techniques or perioperative anxiety were not controlled. The distribution of procedures varied significantly across different types of anesthesia. For example, GA was mostly used for all bariatric and GS procedures, while GA+RA was more common for orthopedic procedures. The type of procedure could influence pain levels and analgesic requirements. Different procedures inherently involve varying levels of tissue trauma and postoperative pain. The duration of surgery is not included in this study because most of the DSU procedures have durations ranging from 1 to 2 hours. 

Strengths

The study’s strengths include its multicenter and prospective design, which reduces bias, enhances generalizability, and allows adjustment for multiple clinically relevant variables (e.g., age, gender, and procedure type). The large sample size (*N* = 395) exceeds the minimum requirements and provides robust statistical power. The study identified female gender, preoperative pain, and lack of LA infiltration as independent predictors of severe pain. Moreover, it emphasized the implementation of important interventions, e.g., procedure-specific protocols, preoperative education, and routine LA infiltration to reduce severe postoperative pain and enhance satisfaction.

*Future Direction:* Research should focus on developing validated risk-prediction models for postoperative pain, testing targeted interventions for high-risk groups, and assessing long-term pain outcomes. Future studies should also explore digital health solutions such as telemedicine for preoperative screening, pain assessments, monitoring, and long-term follow-up. The use of artificial intelligence and machine learning technology to conduct detailed analyses of large data samples on the interplay between patient variables, anesthesia type, surgical procedure, and analgesic needs.

## Conclusions

This study highlights the importance of effective management of postoperative pain in the DSU. The study confirms significant variability in postoperative pain based on the type of procedure, and this is influenced by several patient-specific and intraoperative factors. While aligned with existing literature, some discrepancies highlight the complexity of pain experience and support further investigation into targeted interventions. It emphasizes the need for individualized, procedure-specific, and evidence-based pain management approaches to improve outcomes and patient satisfaction. The clinical implication of our findings highlights some recommendations, including implementation of procedure-specific postoperative pain management, preoperative risk screening, standardized LA infiltration or alternative RA blockade such as preoperative transversus abdominis plane (TAP) blocks, and enhanced preoperative education to improve patient satisfaction and outcomes.
